# Androgen Abuse among Recreational Athletes

**DOI:** 10.1055/s-0039-3401007

**Published:** 2019-12

**Authors:** Maíta Poli de Araujo

**Affiliations:** 1 Department of Gynecology, Escola Paulista de Medicina, Universidade Federal de São Paulo, São Paulo, SP, Brazil

Amateur sports are the most popular form of physical activity in the world. While the media places its attention on professional sports leagues or the Olympic Games, for every professional athlete of a certain sport there are thousands of people who play the same sport to meet their personal needs and fitness requirements.^1^

In women, regular and adequate levels of physical activity improve the muscular and cardiorespiratory systems, reduce the risk of hypertension, coronary heart disease, stroke, diabetes, breast cancer and depression, and are essential to weight control.

However, in order to look thinner and athletic in a short amount of time, the use of performance enhancing substances has increased significantly. Among these substances, androgens are very attractive.

Androgen abuse epidemiology is higher in recreational sportspeople living in Europe, North America (the United States), Oceania (Australia and New Zealand), and South America (Brazil), and lower in Africa and Asia. A recent meta-analysis estimated that the lifetime prevalence of performance substance abuse worldwide is of 18.4% among recreational athletes.^2^

Androgens are hormones that have anabolic properties, with a direct effect on muscle hypertrophy, increased metabolism, and lipolysis. Testosterone is a 19-carbon steroid, and is the most potent endogenous androgen. Anabolic androgenic steroids (AASs) are synthetic compounds that resemble the natural hormone testosterone.^3^

Some athletes use AASs continuously, but others try to minimize their possible adverse effects through *different patterns* of use, as in the following:^4^

- **Cycling**: Users take AASs in cycles of *6 to 12 weeks* followed by 4 weeks to several months off.
- **Stacking**: Users combine several different types of steroids or incorporate other supplements to maximize the effectiveness of the steroids.
- **Pyramiding**: Users gradually increase the dose to a peak, and then reduce the amount.

Androgens and AASs can be taken orally (methandienone, stanozolol, and oxandrolone, for example), as pellets implanted under the skin (pharmaceutical compounding), by injection (nandrolone decanoate and testosterone cypionate, for example), or through the skin as a cream or gel (testosterone). In sports, endogenous AASs, their metabolites and their isomers, when administered exogenously, are prohibited in males and females.^5^ Testosterone may promote athletic performance not only through its long-term anabolic actions, but also by acting on specific substrates in the brain to increase aggression and motivation for competition.

Illicit steroids may be sold at gyms, sporting competitions, and via mail order, and buyers may be at risk of receiving adulterated or contaminated products. Anabolic androgenic steroids are also often illegally sourced from pharmacies or synthesized in clandestine laboratories.^6^

**Fig. 1:**
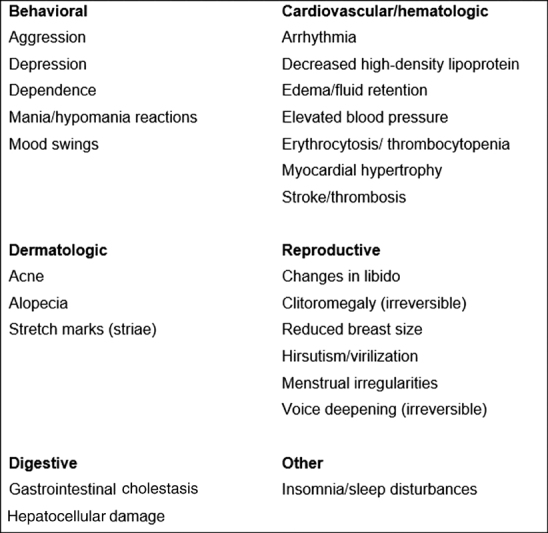
Side effects associated with anabolic steroid abuse in female athletes.

The amateur athletes who most often use anabolic steroids are runners, followed by bodybuilders, cyclists, and weightlifters. The use commonly begins around age 20, and a typical user has at least 1 year of experience training. Most of them are aware of the risks, but believe that the side effects are temporary.^6^ An extensive range of serious side effects is associated with abuse of anabolic steroids (Fig. 1).^7^

The most frequently reported adverse effects among female athletes include menstrual irregularities, clitoromegaly, libido changes, uterine atrophy, hirsutism, alopecia, and deepening of the voice. Adverse effects on the female reproductive system may occur due to depression of gonadotropin release either by direct action on the pituitary gland or by suppression of the hypothalamic GnRH release.^8^

These adverse effects may initiate after months (menstrual disorders, acne, and alopecia, for example), and they can be reversible (alopecia and amenorrhea, for example) or irreversible (voice and clitoris changes, for example).

Several factors, such as the type of sport, exercise intensity, energy balance, fat composition, irregular eating behavior, and emotional stress may contribute to a state of hypogonadotropic hypogonadism in female athletes, even without AAS abuse. However, health professionals and amateur athletes should know that prolonged periods of hypogonadism may lead to severe adverse health consequences, such as effects on mood and memory, lipid abnormalities, low bone mineral density, atherosclerosis, and increased cardiovascular risk.^9^

Animal model studies have shown that some androgens (mainly those injectable) affect the sexual cycle and promote histological alterations in the ovaries and uterus. Destruction of follicular units and an absence of corpus luteum in the ovaries and vacuolated epithelium and endometrial stroma fibrosis of the uterus have been observed. Although there are no controlled studies in humans, these findings are important to guide amateur athletes using anabolic steroids about their reproductive future.^10^

Certainly, each athlete will always be responsible for their body and the decision to use illicit steroids. The same goes for how people will use social media and other technologies. However, the use of AASs seems to involve other characters, such as doctors, digital influencers, bloggers, nutritionists, coaches, and backroom laboratories, and that makes it a social and public health problem.

In conclusion, androgen abuse by recreational athletes is an emerging issue that may need special attention on the part of gynecologists to manage the collateral effects and preserve fertility.
